# Da Artéria Coronária ao Rim: Redefinindo os Objetivos da Intervenção Coronária Percutânea Otimizada por Imagem Intracoronária

**DOI:** 10.36660/abc.20260329

**Published:** 2026-05-29

**Authors:** José Mariani, Stefano Garzon Dias Lemos, Pedro Alves Lemos

**Affiliations:** 1 Hospital Israelita Albert Einstein São Paulo SP Brasil Hospital Israelita Albert Einstein, São Paulo, SP – Brasil; 2 Santa Casa de Misericórdia de São Paulo São Paulo SP Brasil Santa Casa de Misericórdia de São Paulo, São Paulo, SP – Brasil

**Keywords:** Ultrassom Intracoronário, Angioplastia Complexa, Nefropatia Induzida pelo Contraste

A nefropatia associada ao contraste tem sido tratada há muito tempo como uma situação aceitável na cardiologia intervencionista — uma complicação reconhecida, mas raramente priorizada. Por décadas, a área conviveu com a realidade incômoda de que um procedimento cardíaco tecnicamente impecável pode deixar o paciente com uma função mensuravelmente pior em outro órgão — o rim — às vezes de forma permanente. Atualmente, com o diabetes, a hipertensão e a doença renal crônica (DRC) atingindo níveis "pandêmicos", essa resignação não é mais defensável. O determinante modificável mais direto dessa lesão — o volume de contraste — agora pode ser controlado, e o ultrassom intravascular (IVUS) está no centro dessa solução.^1^

A utilização do IVUS em intervenções coronárias complexas está bem estabelecida. Ele redefine a forma como dimensionamos stents, avaliamos a expansão e identificamos subexpansão, má aposição, dissecções de borda e carga residual de placa.^2^ Mas seu valor vai além dos aspectos técnicos da complexidade da lesão: nas mãos certas e com intenção deliberada, o IVUS transforma o mesmo procedimento em um procedimento substancialmente mais conservador para os rins. A ferramenta que otimiza a angioplastia coronária pode, simultaneamente, proteger os rins — desde que optemos por utilizá-la dessa forma.

A base de evidências que sustenta essa dupla função amadureceu consideravelmente. O uso rotineiro de IVUS para guiar angioplastias coronárias é consistentemente empregado para reduzir eventos clínicos em cenários de alta complexidade — doença da Tronco da artéria coronária esquerda, bifurcações, lesões longas, oclusões totais crônicas e síndromes coronárias agudas.^3,4^ Dados paralelos de estudos de redução de volume de contraste confirmam que o volume de contraste pode ser drasticamente reduzido sem comprometer os resultados intervencionistas, mesmo em pacientes com insuficiência renal significativa.^5^ Enquanto isso, as consequências da lesão renal associada ao contraste não são mais meramente bioquímicas: elas se traduzem em maior mortalidade, hospitalizações mais frequentes, maior necessidade de terapia de substituição renal e custos substanciais — mesmo quando as elevações de creatinina parecem modestas.^6–8^ A implicação lógica é difícil de ignorar: continuar expondo pacientes de alto risco a grandes volumes de contraste, quando técnicas de redução do contraste estão disponíveis — e quando a IVUS pode amplificar substancialmente essas reduções — vai além de uma mera escolha técnica e se torna uma questão de responsabilidade profissional. Notavelmente, dados do mundo real do registro DISTRACTION demonstram que essa estratégia é viável como uma abordagem padrão para todos os pacientes — incluindo pacientes com IAMCSSST, choque cardiogênico e DRC — atingindo uma taxa de lesão renal aguda associada ao contraste (LRA-AC) de 1,1% em 4.328 procedimentos consecutivos, mesmo sem orientação IVUS obrigatória devido a restrições do sistema público de saúde.^9,10^

A experiência de centros que realizam intervenção coronária percutânea com contraste mínimo ou nulo em pacientes com DRC avançada confirma a viabilidade dessa abordagem. Os fios-guia podem ser posicionados, as lesões preparadas e os stents implantados e otimizados sob fluoroscopia e imagem intracoronária, com o contraste sendo usado apenas seletivamente — ou omitido completamente — sem comprometer a segurança ou a eficácia.^1,7,8^ Não há atalhos. É necessário domínio avançado do IVUS, planejamento pré-procedimento rigoroso, uma equipe coordenada, protocolos explícitos de conservação de contraste e uma verdadeira mudança cultural na concepção do procedimento desde o início. Metas predefinidas de volume de contraste, registro sistemático das taxas de depuração de contraste/creatinina, disciplina na limitação das angiografias e uso criterioso do mapeamento dinâmico são partes da infraestrutura operacional necessária.

As diretrizes internacionais de revascularização já recomendam o IVUS em cenários de maior complexidade e reconhecem seu impacto nos desfechos clínicos.^9^ Documentos de consenso mais recentes enfatizam estratégias estruturadas para prevenir a lesão renal associada ao contraste, incluindo a limitação do volume de contraste, protocolos de hidratação, seleção do agente de contraste e uso seletivo de imagens intracoronárias.^9^ No Brasil, as diretrizes da Sociedade Brasileira de Cardiologia têm incorporado progressivamente essa perspectiva, porém ainda persiste uma lacuna entre as recomendações e a prática clínica rotineira.

Os autores do artigo "Intervenção Coronária Percutânea com Volume de Contraste Ultrabaixo versus Intervenção Coronária Percutânea Convencional em Pacientes com Insuficiência Renal Pré-Existente: Uma Revisão Sistemática e Metanálise de Desfechos Clínicos"^11^ devem ser parabenizados pelo artigo publicado nesta edição da ABC. Sua contribuição é extremamente bem executada, meticulosamente escrita e transmite a mensagem clara de que a intervenção coronária percutânea com ultrabaixo contraste é "segura, viável e associada a uma redução estatisticamente significativa da lesão renal aguda induzida por contraste". No entanto, essa estimativa deve ser interpretada com cautela, dada a substancial heterogeneidade entre os estudos (I² = 96,7%), o que limita a interpretabilidade de qualquer tamanho de efeito agrupado. Essa descoberta, contudo, precisa ser contextualizada. A LRA-AC é um critério de avaliação substituto. Desfechos clínicos relevantes, como mortalidade, necessidade de diálise e eventos cardiovasculares adversos maiores (MACE), não atingiram significância estatística, com intervalos de confiança muito amplos para permitir quaisquer conclusões. Além disso, o desfecho composto de MACE não foi decomposto em seus componentes individuais, o que impede a identificação de qual elemento, se houver, impulsiona a tendência observada. Ademais, a metarregressão que relata R² = 100% com seis estudos (quatro graus de liberdade residuais) representa um artefato matemático, e não uma descoberta explicativa robusta. Essas limitações são intrínsecas e esperadas em uma área onde as evidências randomizadas ainda são escassas.

E a mensagem chega em um momento relevante: reforça caminhos concretos e viáveis para uma cardiologia intervencionista que leva a sério o risco renal sem abrir mão da qualidade técnica.

O desafio que temos pela frente não é apenas clínico, mas também estrutural: traduzir o conhecimento em compromisso institucional, tornar a lesão renal associada ao contraste um alvo explícito de prevenção em todos os laboratórios de cateterismo e defender políticas que promovam esses objetivos. Tecnologias ao alcance de todo o sistema de saúde.
