# Supplementary material for: Da Artéria Coronária ao Rim: Redefinindo os Objetivos da Intervenção Coronária Percutânea Otimizada por Imagem Intracoronária
Source: Arq Bras Cardiol. 2026 May 29;123(4):e20260329. [Article in Portuguese] doi: 10.36660/abc.20260329 (PMC13398866; doi:10.36660/abc.20260329)
Supplement: Translation

# Translation: From Coronary Artery to Kidney: Redefining the Goals of Intracoronary Imaging–Optimized Percutaneous Coronary Intervention

**DOI:** 10.36660/abc.20260329i

**Authors:** José Mariani, Stefano Garzon Dias Lemos, Pedro Alves Lemos

**Affiliations:** 1 São Paulo SP Brazil Hospital Israelita Albert Einstein, São Paulo, SP – Brazil; 2 São Paulo SP Brazil Santa Casa de Misericórdia de São Paulo, São Paulo, SP – Brazil

**Keywords:** Intracoronary Ultrasound, Complex Angioplasty, Contrast-Induced Nephropathy

Contrast-associated nephropathy has long been treated as an acceptable trade-off in interventional cardiology — a complication acknowledged but rarely prioritized. For decades, the field has lived with the uncomfortable reality that a technically flawless cardiac procedure can leave a patient with measurably worse function in another organ – the kidney – sometimes permanently. In current days, when diabetes, hypertension, and chronic kidney disease (CKD) are reaching "pandemic" levels, that resignation is no longer defensible. The most direct modifiable determinant of this injury — contrast volume — is now addressable, and intravascular ultrasound (IVUS) sits at the center of that solution.^1^

The case for IVUS in complex coronary intervention is well established. It redefines how we size stents, assess expansion, and identify underexpansion, malapposition, edge dissections, and residual plaque burden.^2^ But its value extends beyond the technicalities of lesion complexity: in the right hands and with deliberate intent, IVUS transforms the same procedure into a substantially more renal-sparing one. The tool that optimizes coronary angioplasty can simultaneously protect the kidney — provided we choose to use it that way.

The evidence base supporting this dual role has matured considerably. Routine IVUS to guide coronary amphiplasties is consistently used to reduce clinical events across high-complexity scenarios — left main coronary artery, bifurcations, long lesions, chronic total occlusions, and acute coronary syndromes.^3,4^ Parallel data from contrast volume reduction studies confirm that contrast volume can be dramatically decreased without compromising interventional outcomes, even in patients with significant renal impairment.^5^ Meanwhile, the consequences of contrast-associated kidney injury are no longer merely biochemical: they translate into higher mortality, more frequent hospitalizations, increased need for renal replacement therapy, and substantial costs — even when creatinine elevations appear modest.^6–8^ The logical implication is hard to escape: continuing to expose high-risk patients to large contrast volumes, when contrast-sparing techniques are available — and when IVUS can substantially amplify these reductions — moves beyond a mere technical choice and toward a matter of professional responsibility. Notably, real-world data from the DISTRACTION registry demonstrate that this strategy is feasible as a default approach for all-comers — including STEMI, cardiogenic shock, and CKD patients — achieving a contrast-associated acute kidney injury (CA-AKI) rate of 1.1% across 4,328 consecutive procedures, even without mandatory IVUS guidance due to public health system constraints.^9,10^

Experience from centers performing minimal- or zero-contrast percutaneous coronary intervention in advanced CKD confirms the feasibility of this approach. Wires can be positioned, lesions prepared, and stents deployed and optimized under fluoroscopy and intracoronary imaging guidance, with contrast used only selectively — or omitted altogether — without sacrificing safety or efficacy.^1,7,8^ There is no shortcut. It demands advanced IVUS proficiency, rigorous pre-procedural planning, a coordinated team, explicit contrast-conservation protocols, and a genuine cultural shift in how the procedure is conceived from the get-go. Predefined contrast-volume targets, systematic recording of contrast-to-creatinine clearance ratios, discipline in limiting angiographic runs, and judicious use of dynamic roadmapping are all part of the operational infrastructure required.

International revascularization guidelines already recommend IVUS in higher-complexity scenarios and acknowledge its impact on clinical outcomes.^9^ More recent consensus documents emphasize structured strategies to prevent contrast-associated kidney injury, including contrast-volume limitation, hydration protocols, contrast agent selection, and selective use of intracoronary imaging.^9^ In Brazil, the *Sociedade Brasileira de Cardiologia*'s guidelines have progressively incorporated this perspective, yet a gap between recommendations and routine practice persists.

The authors of the article "Ultra-Low Contrast versus Conventional Percutaneous Coronary Intervention for Patients with Baseline Renal Impairment: A Systematic Review and Meta-Analysis of Clinical Outcomes"^11^ must be congratulated for their article in this issue of ABC. Their contribution is extremely well executed, meticulously written, and lands the clear message that ultra-low contrast percutaneous coronary intervention is "safe, feasible, and associated with a statistically significant reduction of contrast-associated acute kidney injury." However, this estimate must be interpreted with caution, given the substantial between-study heterogeneity (I² = 96.7%), which limits the interpretability of any single pooled effect size. This finding must nonetheless be contextualized. CA-AKI is a surrogate endpoint. Hard clinical outcomes, i.e., mortality, dialysis requirement, and major adverse cardiovascular events (MACE), did not reach statistical significance, with confidence intervals too wide to permit any conclusions. Additionally, the MACE composite was not decomposed into its individual components, precluding identification of which element, if any, drives the observed trend. Moreover, the meta-regression reporting R² = 100% with six studies (four residual degrees of freedom) represents a mathematical artefact rather than a robust explanatory finding. These limitations are intrinsic and expected in a field where randomized evidence remains scarce.

And the message arrives at a relevant moment: it reinforces concrete, actionable pathways toward an interventional cardiology that takes renal risk seriously without conceding technical quality.

The challenge ahead is not only clinical but structural — translating knowledge into institutional commitment, making contrast-associated kidney injury an explicit prevention target in every catheterization laboratory, and advocating for policies that bring these technologies within reach of the entire health system.
